# Scope of practice of optometrists working in the UK Hospital Eye Service: Second national survey

**DOI:** 10.1111/opo.12952

**Published:** 2022-02-12

**Authors:** Patrick J G Gunn, Rosalind C Creer, Michael Bowen, Cindy Tromans, Andrew Jonathan Jackson, Andrew P Tompkin, Robert A Harper

**Affiliations:** ^1^ Manchester Royal Eye Hospital Manchester University NHS Foundation Trust Manchester UK; ^2^ Faculty of Biology, Medicine and Health University of Manchester Manchester UK; ^3^ The College of Optometrists London UK; ^4^ Royal Victoria Hospital Belfast Health and Social Care Trust Belfast UK; ^5^ Dublin Technological University Dublin Ireland; ^6^ St Paul's Eye Unit Liverpool University Hospitals NHS Foundation Trust Liverpool UK; ^7^ Liverpool Business School Liverpool John Moores University Liverpool UK

**Keywords:** hospital optometry, independent prescribing, optometry, scope of practice, survey

## Abstract

**Purpose:**

As the landscape in ophthalmology and related commissioning continues to change, there is a pressing need to re‐evaluate the current scope of practice of hospital optometrists working within secondary care in the UK. We aim to establish if the skills or services delivered by optometrists have changed to meet varying demands, and to better understand what changes in practice may have arisen as a result of COVID‐19.

**Method:**

A survey developed from that used in 2015 was disseminated to 129 optometry Hospital Eye Service (HES) leads in September 2020, including questions on department workforce; core services; extended roles; procedures undertaken within extended roles; level of autonomy; arrangements for prescribing; training and accreditation, and service changes in response to COVID‐19.

**Results:**

Ninety responses were received (70% response rate) from within England (76%), Scotland (22%) and Northern Ireland (2%). Whole time equivalents within units ranged from 0.4–79.2 (median of 2.5). In comparison to the 2015 survey, there was an increase in the proportion of units delivering extended roles, with glaucoma (88%) remaining the most common extended role, and new areas of practice in uveitis (21%) and vitreoretinal (13%) services. There was increased use of independent prescribing (67%) in comparison to 18% in 2015 and there was an increase in optometrists delivering laser interventions. In response to COVID‐19, optometrists were increasingly delivering telephone consultations and there were new collaborations between primary and secondary care.

**Conclusions:**

Optometrists’ scope of practice continues to develop in the HES with an increased variety of roles and an apparent increase in the number of units employing optometrists, often working in roles historically performed by medical practitioners. Such changes appear necessary in recovery and transformation within ophthalmology, alongside wider optometry changes arising at the interface of primary and secondary care.

**Key points**

- Optometric scope of practice in the UK Hospital Eye Service continues to expand in terms of the number of departments, optometrists undertaking extended roles and in the variety of sub‐specialties.
- There is an increase in the use of independent prescribing and the number of optometrists delivering laser interventions when comparing UK national survey findings in 2015 and 2020.
- The evolving scope of practice of optometrists continues to enhance the secondary care ophthalmology workforce, as required to help capacity meet demand, and alongside wider pathway changes in primary care.

## INTRODUCTION

In 2016, Harper et al. published the first comprehensive scope of practice survey of hospital optometrists in the UK,^1^ documenting the roles of hospital optometrists which were understood to have developed considerably over the previous two decades or more. The national survey showed that 96% of Hospital Eye Service (HES) optometry departments had optometrists undertaking extended roles, with glaucoma being the leading extended role service, with macula, medical retina/diabetes, cataract and corneal services being the next reported to be commonly performed. A significant degree of autonomy was reported for optometrists working in these clinics, with just 23% of extended role clinics stated to not go ahead without a consultant ophthalmologist present. Ophthalmology has been the busiest specialty in National Health Service (NHS) outpatient care for a number of years.^2^ Hospital optometrists, amongst other eye health professionals, have been highlighted as an important part of the workforce to help meet increasing demand for capacity.^3^, ^4^, ^5^ A wide variety of clinical procedures or interventions were reported to be undertaken, and with a small number of optometrists undertaking specific laser procedures, including selective laser trabeculoplasty (SLT). Following publication of the LiGHT trial,^6^ the National Institute for Health and Care Excellence (NICE) produced an exceptional review of the 2017 guideline for glaucoma, indicating there may be a need to further upskill more non‐medical staff to deliver SLT. The action on age‐related macular degeneration (AMD) group published reports in 2012 and 2013 about the need for all providers with AMD treatment services to evaluate their services and to consider the use of non‐medical professionals (NMPs) to support their services.^7^, ^8^, ^9^ In the 2015 scope of practice survey, whilst there were 71% of optometry departments contributing to medical retina clinics, there were just three departments where optometrists were delivering intravitreal injections for AMD. More recently, the Royal College of Ophthalmologists’ AMD document, The Way Forward (2017), made recommendations for service providers to consider non‐ophthalmologist injectors.^10^

In March 2020, the Coronavirus (COVID‐19) pandemic led to routine NHS hospital outpatient appointments being suspended.^11^ Whilst the impact of COVID‐19 on NHS outpatient waiting lists is not yet fully clear, a retrospective analysis in one department comparing activity in June 2019 (pre‐COVID‐19) and June 2020 (during COVID‐19 pandemic) showed a 63% reduction in outpatient activities.^12^

With demand in ophthalmology increasing due to population aging, as well as the emergence of new treatments associated with a high demand on outpatient services, there is a pressing need to re‐evaluate the current scope of practice of hospital optometrists working within multidisciplinary teams in secondary care in the UK. The aim is to establish if the skills or services delivered by optometrists have changed since 2015 to meet these evolving demands, and to understand what changes in hospital optometry practice there may have been as a result of COVID‐19.

## METHODS

To update the information previously collected in 2015 regarding roles of optometrists within UK hospital optometry, the present survey was developed by senior hospital optometrists and the Director of Research at The College of Optometrists (MB). The survey was based on that implemented in 2015 to permit comparison of change, and specific areas were expanded (noted in points 5. and 6. below) to better evaluate the evolution of hospital optometry services occurring within the 5 years since the first survey.

The final survey included questions covering the following areas:

1. The number of whole‐time equivalent (WTE) optometrists working in the department, the type of hospital this department is based in (e.g., Major Teaching Hospital/ Acute Trust or District General Hospital) and location within the UK.
2. The core service provision, i.e., the roles that would have traditionally been provided by optometrists. Examples include refraction, contact lens services, low vision assessment, biometry, topography.
3. Extended role provision, i.e., those roles that were deemed to fall outside the “traditional” roles of the optometrist as per the 2015 data, including, for example: cornea; glaucoma; medical retina; diabetic eye disease; eye casualty; paediatric assessment; laser/refractive surgery and cataract services, amongst others. Whilst the term “extended role” may be considered outdated, given the longstanding presence of optometrists working in clinics such as glaucoma, for consistency we will use the same terminology for this paper. Additional information was requested about any procedures that were undertaken as part of these extended role clinics (e.g., antiVEGF or other intravitreal injections, laser procedures, bleb manipulations, suture removal, punctal plug insertion/removal, etc). Details were requested about the level of autonomy optometrists held while working within these clinics, as well as arrangements for prescribing medication where required.
4. Training and accreditation; with respect to each of the clinics involving extended roles, information was requested for any requirements for the optometrist to undertake additional training along with the details of the approaches taken. For example, “traditional apprentice style” learning, the use of a logbook, the requirement for specific higher qualifications accredited by The College of Optometrists or other education providers, and/or an internal sign off for competencies.
5. Specific information regarding glaucoma lasers undertaken by optometrists was requested, to establish if optometrists were currently undertaking these procedures, or, if departments had plans for optometrists to undertake this role.
6. Due to the ongoing COVID‐19 pandemic at the time of the survey, an additional question was asked to establish whether any changes in practice due to the pandemic had been implemented, and if so, whether these changes were likely to be incorporated within future service provision.

Further details of the questions and possible response options are provided in Appendix 1.

The SurveyMonkey online survey provider (surveymonkey.com) was used to disseminate the survey to heads of departments/senior optometrists within the HES. A database compiled and maintained by the Hospital Optometrists Committee was used to establish the optometry heads cohort, thereby allowing dissemination of the survey to one primary contact at each HES department known to employ optometrists. For the 2020 survey, hospitals with ophthalmology services were identified from public domain information to generate a new database. Hospitals were identified within each country and NHS region, and their NHS websites checked individually for listed ophthalmology services, with this information being corroborated with other open access sources (e.g., General Optical Council [GOC] register).

A total of 129 hospital optometry department leads were invited to participate (105 in England, 4 in Northern Ireland, 15 in Scotland and 5 in Wales). Initial survey links were circulated in September 2020, with reminders being sent approximately 6, 12 and 15 weeks thereafter. The responses were anonymised, with the authors being unable to establish any links to specific respondents with their responses or comments.

The University of Manchester ethics screening tool^13^ and the NHS Health Research Authority (HRA) ethics screening checklist^14^ confirmed that formal ethical approval was not required. The project was reviewed and approved by the Association of Optometrists (AOP) Hospital Optometrists Committee and The College of Optometrists’ Research Committee. As all email addresses were anonymised and no further personal information was collected or processed; no further ethical review was sought.

## RESULTS

### Demographics

Of the 129 survey invitations disseminated, 90 responses were received from 90 HES departments, reflecting a response rate of 70%. From the 90 responses, 44 (49%) described their department as being part of a Major Teaching Hospital or Acute Trust, 41 (46%) from a District General Hospital and 5 (6%) from community or other hospitals, which was comparable to the 2015 survey. A majority of respondents were based in England (*n* = 68, 76%), with 20 departments (22%) responding from Scotland, two (2%) from Northern Ireland and none from Wales.

The number of WTE optometrists per department ranged from 0.4 to 79.2 and the median calculated was 2.5. Most departments had fewer than 10 optometrists, with only 11 departments (12%) having 10 or more WTE optometrists, compared to eight departments in 2015.

### Core clinical services

**FIGURE 1:**
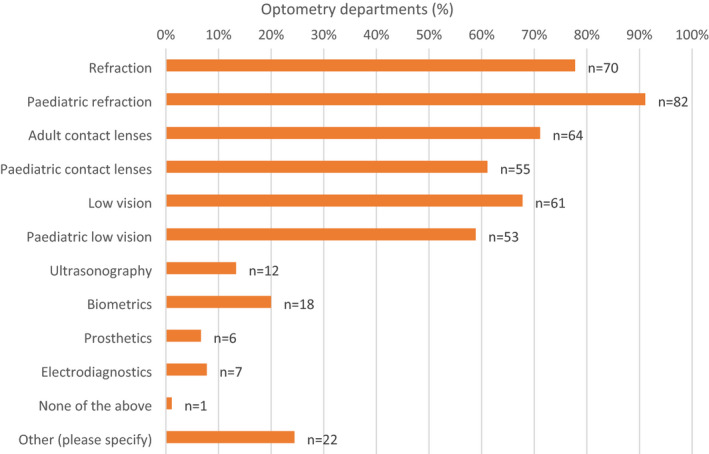
Bar chart illustrating the distribution of respondents (*N* and %) providing the various core clinical services in Hospital Eye Service (HES) departments: “Which core clinical services do optometrists provide at your hospital and/or your peripheral clinics?”

Figure 1 shows the responses to the question regarding the types of services provided within optometry departments (see Appendix 1), where we have classed core clinical services/traditional optometry roles in line with the 2016 paper. Most departments provide refraction, contact lenses and low vision services to both children and adults. The additional services such as biometry, electro‐diagnostics and ultrasonography appear to be provided by <20% of departments. One respondent (1%) stated that they did not provide any of the above services and the “other” category included mention of perimetry, but otherwise more extended roles.

**FIGURE 2:**
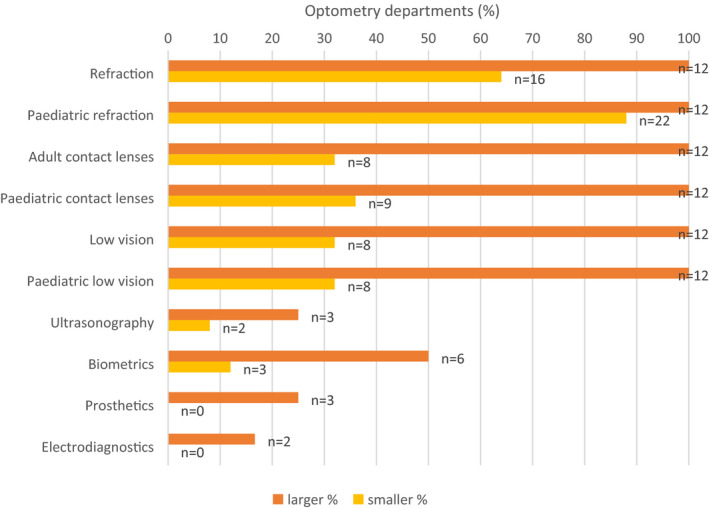
Comparison of hospital departments undertaking specific clinical roles, with larger departments (≥10 WTE optometrists, *N* = 12) and smaller departments (<2 WTE optometrists, *N* = 25): “Which core clinical services do optometrists provide at your hospital and/or your peripheral clinics?” WTE: whole‐time equivalent

In view of the distribution of the number of WTE within departments, a sub‐group analysis was performed comparing ‘larger’ departments to ‘smaller’ departments to assess for any differences in the type of clinics and/or procedures being undertaken by optometrists. The difference between core clinical services offered in smaller and larger departments is detailed in Figure 2, indicating more comprehensive core services provision in the largest departments in comparison to those departments with fewer optometrists.

### Extended roles

**FIGURE 3:**
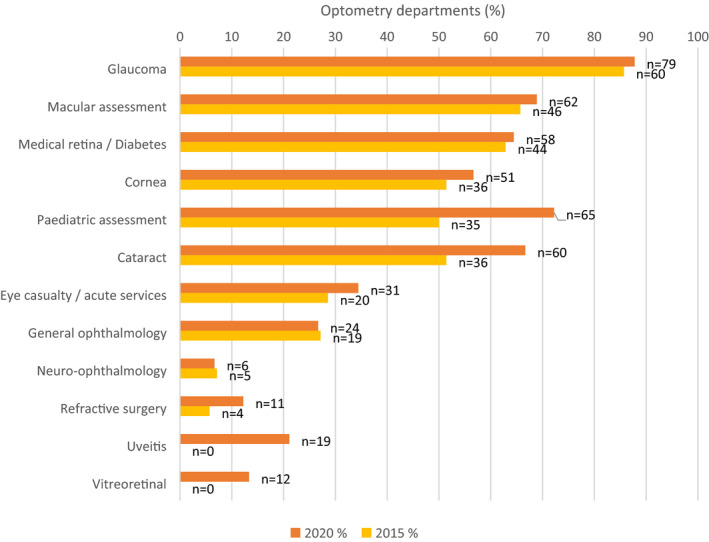
Bar chart comparison of the percentage of hospital optometry departments providing extended roles in subspecialties as labelled above: “Beyond the core optometry services listed in question 4, in which sub‐specialty areas do optometrists provide extended roles at your hospital and which type of patients are seen?”

Figure 3 shows a majority of optometry departments have optometrists undertaking extended roles in glaucoma, with assessments taking place for both new and follow up patients. Macular assessment, medical retina/diabetes, paediatric assessment and cataract services are also subspecialties with a high proportion of departments stating optometrists are involved in the provision of extended roles. Neuro‐ophthalmology appears to be the specialty with the fewest optometrists providing support within the service. Subspecialties listed within the “other” section include paediatric uveitis, specialist craniofacial, and clinics for patients with learning disabilities, although no specific details were given for what level of assessment was being provided.

### Autonomy and extended roles

**FIGURE 4:**
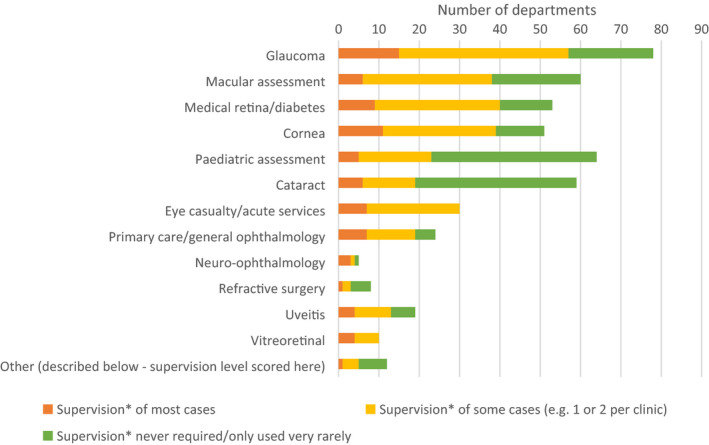
Bar chart illustrating responses for the question on supervision of cases: “When considering each of the options selected in question 5, what level of autonomy do optometrists have when managing cases within those clinics? (Note context is in relation to optometrists who are fully trained within that service)”

Overall, more respondents stated that supervision is required occasionally (47%), followed by rarely or not at all (37%), with just 17% of respondents stating supervision is required in all cases. However, the autonomy of optometrists within these extended roles varies between subspecialty (see Figure 4). Paediatric assessment and cataract clinics appear to receive the least amount of supervision, although this supervision may vary depending on what type of practice is being undertaken within these subspecialties. There was an increase in respondents reporting that supervision was either never or rarely present in glaucoma (*n* = 21, 26.9%), macular assessment (*n* = 23, 36.7%), medical retina/diabetes (*n* = 13, 24.5%) and corneal clinics (*n* = 12, 23.5%) when compared to the previous survey, where no respondent reported rare/no supervision in these areas.

### Prescribing of medications

**FIGURE 5:**
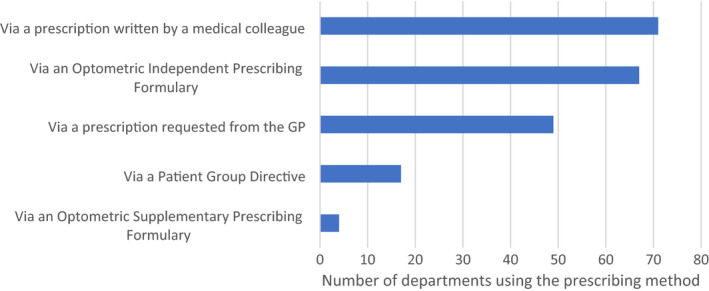
Use of prescribing methods: “If the patient requires a medical prescription following optometry assessment in clinic, how is this prescribed?”. Responses are in relation to all prescribing options. GP, General Medical Practitioner

Figure 5 shows the most common methods by which medications, where necessary, are prescribed, and shows that prescriptions written by a medical colleague and those written by independent prescribing optometrists are the more popular forms for prescribing. The latter figure shows an increase in the use of independent prescribing compared to the previous survey (i.e., an increase to 67% using independent prescribing as a method of prescribing from 18% in the 2015 survey). Independent prescribing was the most common method of prescribing for 46% of departments.

**FIGURE 6:**
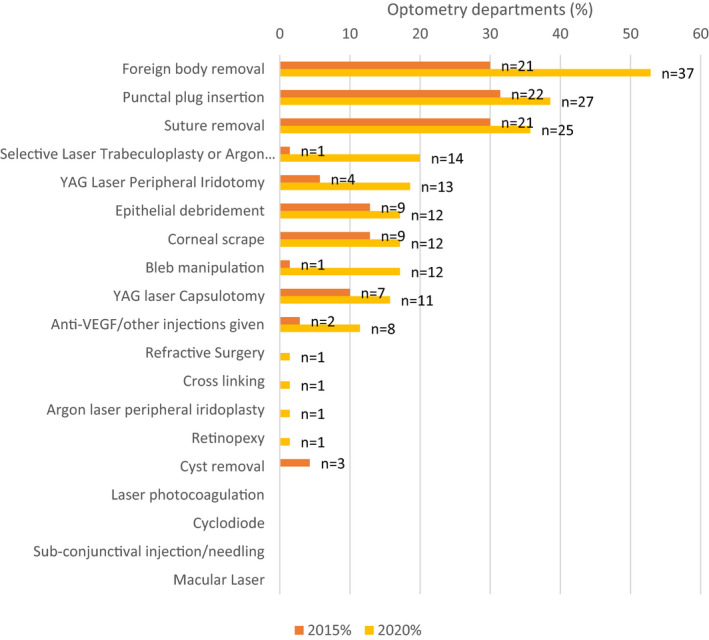
Bar chart illustrating a comparison between procedures stated to be undertaken by optometrists from respondents in the 2020 and 2015 surveys. The question asked: “Are any of the following procedures undertaken by optometrists working within these services?”. Laser YAG capsulotomy was inadvertently omitted from the list in the 2020 questionnaire; therefore these data were added by respondents in the “other” category and may reflect an under‐estimate

### Procedures undertaken within extended roles

Hospital optometry leads were asked about specific procedures undertaken by optometrists as part of their extended roles (Figure 6). Foreign body removal was the most common procedure, followed by suture removal and corneal epithelial debridement. No hospital optometry department reported optometrists performing cyclodiode laser, pan‐retinal photocoagulation, macular laser or eyelid cyst removal.

When asked if service leads were considering introducing optometrists to perform these procedures, 14 respondents stated they would be expecting optometrists to undertake selective laser trabeculoplasty and 11 for Yttrium Aluminum Garnet (YAG) laser peripheral iridotomy in the next 12 months. Between eight and 10 departments are planning for optometrists to start undertaking procedures such as foreign body removal, corneal scrapes and suture removal within the next 12 months.

### Training and accreditation

**FIGURE 7:**
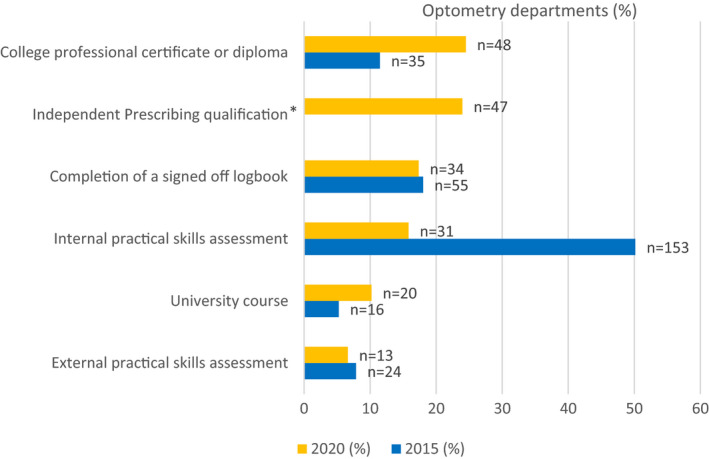
Bar chart illustrating methods employed by departments for accreditation in one or more of the clinical roles. Data from 2015 are compared to data from 2020. Accreditation responses were sought for *each* sub‐specialty and the numbers of responses combined. (*Note, for the independent prescribing qualification, data from 2015 are unavailable)

Training and accreditation arrangements were explored to gain insight into the approaches used when implementing these extended roles. Apprentice style approaches to training, including both observation sessions and sessions worked alongside those taking a supervisory role, were popular methods, with 64% of departments stating this approach was used for all clinical roles. For accreditation, there is a greater use of professional qualifications compared to the previous survey, with the use of logbooks and internal practical skills assessment used regularly (Figure 7).

### The impact of the COVID‐19 pandemic

**FIGURE 8:**
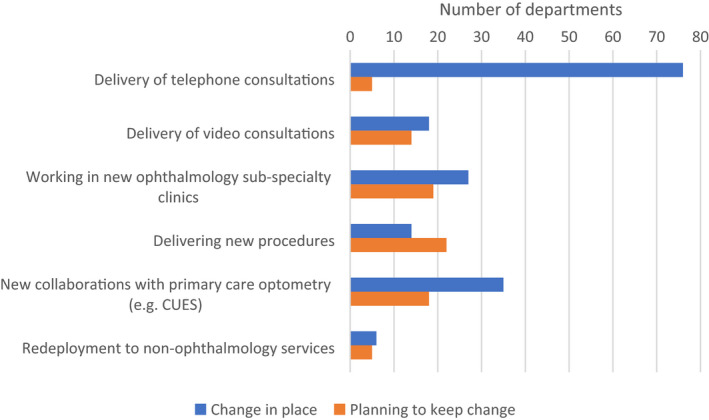
Changes in roles of optometrists due to the COVID‐19 pandemic: “How has the COVID‐19 pandemic impacted the role of optometrists working in your department?”

The major difference in optometric practice reported by respondents because of COVID‐19 was the increased use of remote telephone and video consultations (Figure 8). More optometry departments were planning to continue to use remote video consultations versus telephone consultations post‐pandemic. In relation to extended roles, optometrists appear to be taking on new roles and new procedures to help when other staff were re‐deployed, and it appears that most of these roles will continue or increase in the future. Some respondents noted new collaborations with primary care optometry during the pandemic, some of which were considered likely to continue beyond recovery, and some of which involve engagement of secondary care optometric staff at the interface of primary to secondary care pathways.

## DISCUSSION

In this second national survey of hospital optometrists, there was an increase in the number of hospitals with optometry departments from 79 in 2015 to 129 in 2020. We obtained a greater number of responses (90) than in the 2015 survey published in 2016^5^ (70 responses). Whilst the overall response rate was higher in the earlier survey (89% in 2015 versus 70% in 2020), it might have been affected by the challenging circumstances of COVID‐19 and may be argued to reflect a good response rate. The median number of WTE optometrists was slightly reduced from 3.3 in 2015 to 2.5 in 2020, and this apparent shift may relate to the larger number of ophthalmology units establishing optometry departments beyond that of larger units expanding their existing optometry workforce. However, it is clear that some of the larger optometry departments are expanding, since the upper range of WTEs increased from around 60 to almost 80, with a small increase in the number of departments having greater than 10 WTE within the team. As was the case in 2015, most departments had fewer than 10 WTE optometrists.

The results of this scope of practice survey show most units with greater than 10 WTE hospital optometrists continue to deliver a comprehensive range of traditional core optometry services including refraction, low vision and contact lenses, both to adults and children. Some departments still have optometrists performing biometry, electro‐diagnostics, prosthetics or perimetry, but in no more than 20% of departments. Across these historically labelled traditional or core optometry services, the proportion of departments offering these services was less than in the previous survey. Indeed, optometrists have been working in subspecialty areas of ophthalmology such as glaucoma, medical retina and cataract for many years, and it can be argued that there is a shift in what should be considered core hospital optometrists’ roles, i.e., to those set out in the previous survey. In terms of the comparison between small and larger optometry departments, it is apparent that larger units tend to offer a more comprehensive range of core optometry services, with more variability in the proportion of core services provided in smaller units.

The overall proportion of departments with optometrists in extended roles in the varying subspecialties has not changed dramatically, despite the number of respondents and the WTE of those working in extended roles increasing. However, notwithstanding differences in the sample size, the potential for responses from new units and the response rate across the two timelines, comparing the present survey results to the previous one shows an increase in extended roles in all sub‐speciality areas. Indeed, the specific sub‐specialities with the higher numbers of departments delivering these remain similar. Glaucoma remains the extended role where hospital optometrists are most likely to be involved, followed by macular assessment. Whilst in 2015, no departments reported optometrists working in uveitis, this figure has now increased to 20. Responding departments from the last survey also showed a lack of optometrists working within vitreoretinal clinics in 2015, and this figure has now increased to 12, including one department where optometrists deliver retinopexy, indicating extension in scope of practice by optometrists working in these sub‐specialties. Perhaps surprisingly, the proportion of departments with optometrists working in acute ophthalmology appeared to be stable during this period. The decline in numbers of optometrists working in general ophthalmology clinics likely relates to the growth in subspecialty clinics in the HES^15^ versus general ophthalmology.

There has been an increase in the number of departments where optometrists have independent prescribing as an option, and this change is likely to have facilitated increased autonomy for optometrists working in these clinics. Approximately 25% of respondents stated that supervision was rarely required in glaucoma, macular, medical retina/diabetes and corneal clinics, as compared to the previous survey where no respondents selected this option to describe supervision in these subspecialty areas. In 2020, Greenwood et al. published a snapshot survey which included data concerning scope of practice for ophthalmic nurses, orthoptists and optometrists within 34 ophthalmic departments, mainly in England.^16^ Interestingly, our results regarding supervision of cases from the present survey differs from the work of Greenwood et al., since they highlighted supervision levels to be high for optometrists in cataract services. Our results showed cataract services to be the extended role where optometrists were more likely to never/almost never need supervision. It is possible that this discrepancy is a sampling issue, although assumptions around definitions of supervision may have contributed to differences in responses to the respective surveys.

Since the 2015 survey, the number of hospital departments reporting optometrists delivering glaucoma‐related laser interventions has increased from four departments to 13 delivering YAG peripheral iridotomy, and from one to 14 delivering selective laser trabeculoplasty. There were nine departments planning to deliver YAG peripheral iridotomy and 14 planning to deliver selective laser trabeculoplasy within the next 12 months. Whilst the numbers of optometrists performing glaucoma post‐surgical bleb manipulation is still low, these numbers have increased, with just one respondent in the 2015 survey stating they were using bleb manipulation, contrasting in 2020 with 12 respondents reporting use of bleb massage and with three respondents reporting optometrists perform bleb needling. At the same time, the reported level of supervision has decreased in glaucoma clinics from supervision of most cases in the previous survey being the most likely response to only occasional supervision in 2020, despite one respondent reporting an increase in complexity of cases due to COVID‐19, and therefore an associated increase in supervision. Further qualitative research surrounding autonomy of optometrists within subspecialty areas may be beneficial.

Regarding other laser procedures, at least 11 departments (12.2%) reported optometrists to be delivering YAG capsulotomy in 2020 compared to seven in the previous survey (8.9%), although this figure is likely to be an underestimate, since this specific category had been inadvertently omitted as a specific option from our questionnaire.

In macular assessment clinics, there has been an increase in the number of departments where optometrists are delivering intravitreal injections, increasing from three departments in 2015 to eight departments in 2020; however, the numbers of optometry departments delivering intravitreal injections remains low, which is in agreement with the survey by Greenwood et al.,^16^ where they found nurses were most likely to deliver such injections.

Apprentice style training remains an important method of training, as was the case in the previous survey, but there was an increase in the use of the College of Optometrists’ professional certificates/diploma as the method of accreditation, with 53.3% of respondents using this as a method of accreditation in at least one extended role. This increase may relate to the increased availability and/or accessibility of these qualifications, and/or guidance for the need for health care professionals to have such a professional qualification in areas such as glaucoma.^17^ Despite the diversity of clinical roles expanding in 2020 (e.g., uveitis and vitreoretinal), in comparison to 2015, it appears that there is proportionately less reliance on internal practical skills assessment and more reliance on College professional qualifications. Arguably, with extended roles encompassing a broader range of subjects without a related recognised higher qualification, one may have expected a greater reliance on local accreditation methods. We acknowledge that our 2020 survey did not capture subspecialist training and accreditation methods, and so we are not able to comment on the relative differences that may exist between requirements in different extended roles.

When asked about changes in practice resulting from COVID‐19, many respondents reported telephone and video consultations being used. Whilst this finding was perhaps unsurprising given the widespread use of remote consultations in medicine during COVID,^18^, ^19^, ^20^ the numbers of departments planning to continue use of such provisions was quite low. Although there is some data on patient and clinician experience in video consultations in ophthalmology,^21^ there is a relative paucity of literature in this area, with service providers potentially lacking confidence that this method of service delivery will be effective in the recovery phase of COVID. Some respondents confirmed that their hospital trusts had newly established services with primary care optometry during the COVID‐19 pandemic. It is not clear from the data what involvement hospital optometrists may have in implementing these new services, but as an example, the Manchester COVID Urgent Eyecare Service (CUES)^22^ included hospital optometrists in planning, implementing and delivering this service. A much higher proportion of those delivering such new services planned to continue with them into the future, and perhaps innovations such as digital image transfer used in CUES may be a more effective use of technology, rather than video consultations directly with patients. Since there were fewer respondents stating that optometrists were delivering new procedures (as a result of COVID‐19) than those who stated they planned to keep this change in place, these specific questions may have been interpreted incorrectly by some respondents.

This paper reports on the second national survey of the scope of practice of optometrists in the UK HES published within the peer review literature, and provides an interesting comparison to the previous survey^1^ in demonstrating evolution of scope of practice over time. Unfortunately, there were no responses from the HES units surveyed in Wales, a country with well‐established enhanced community services^23^ and only a low number of departments with optometrists (5) having been invited to participate. Our results therefore reflect practice in three nations within the UK, i.e., Scotland, Northern Ireland and England. The focus of this paper was on the scope of practice of hospital optometrists. However, there has been a drive, both before and during the COVID‐19 pandemic, to move a proportion of patients from secondary to primary care.^24^, ^25^ Further work looking at the scope of practice of primary care optometrists working in community practice as well as the distribution and availability of those with relevant professional qualifications would be beneficial to determine what provision might become available where there are gaps in services which need addressing.

In conclusion, the scope of practice of optometrists in the UK HES continues to evolve such that hospital optometry now embraces a growing range of service contributions well beyond the traditional areas. Indeed, one could argue that the concept of “core” hospital optometry needs updating. Comparison of the 2015 and 2020 surveys illustrates a strengthening of contributions in glaucoma care and novel contributions to other ophthalmic sub‐specialties. There is a growing cohort of optometrists engaged in the provision of laser interventions, within an allied framework of growing autonomy and independence, as befits clinicians with knowledge, training and accreditation, and experience with the relevant case mix. The change in practice demonstrated here was evolving even pre‐pandemic, in an attempt to meet the already growing demand for capacity in ophthalmology. Indeed, it seems likely that eyecare pathways will require further scope of practice changes within optometry. Arguably, the present survey illustrates that the change occurring organically over time for the optometric workforce within secondary care shows promise for the growing potential for further primary care optometric engagement in managing the ocular disease burden currently faced by the HES.

## CONFLICT OF INTEREST

The authors report no conflicts of interest and have no proprietary interest in any of the materials mentioned in this article.

## AUTHOR CONTRIBUTIONS

**Patrick J G Gunn:** Conceptualization (equal); Data curation (equal); Formal analysis (equal); Funding acquisition (equal); Investigation (equal); Methodology (equal); Project administration (lead); Writing – original draft (lead); Writing – review & editing (lead). **Rosalind C Creer:** Conceptualization (supporting); Data curation (equal); Formal analysis (equal); Funding acquisition (supporting); Investigation (equal); Methodology (supporting); Project administration (equal); Validation (equal); Writing – original draft (equal); Writing – review & editing (equal). **Michael Bowen:** Conceptualization (equal); Data curation (equal); Formal analysis (supporting); Funding acquisition (lead); Investigation (supporting); Methodology (equal); Project administration (supporting); Resources (equal); Software (lead); Writing – original draft (supporting); Writing – review & editing (supporting). **Cindy Tromans:** Conceptualization (equal); Data curation (supporting); Formal analysis (supporting); Funding acquisition (supporting); Investigation (supporting); Methodology (equal); Project administration (supporting); Writing – original draft (supporting); Writing – review & editing (supporting). **Andrew Jonathan Jackson:** Conceptualization (supporting); Data curation (supporting); Formal analysis (supporting); Funding acquisition (supporting); Investigation (supporting); Methodology (equal); Project administration (supporting); Writing – original draft (supporting); Writing – review & editing (supporting). **Andrew P Tompkin:** Conceptualization (supporting); Data curation (equal); Formal analysis (supporting); Funding acquisition (supporting); Investigation (supporting); Methodology (supporting); Project administration (equal); Resources (equal); Writing – original draft (supporting); Writing – review & editing (supporting). **Robert A Harper:** Conceptualization (lead); Data curation (supporting); Formal analysis (equal); Funding acquisition (equal); Investigation (equal); Methodology (equal); Project administration (supporting); Supervision (lead); Writing – original draft (equal); Writing – review & editing (equal).
